# The Effect of RNA Substitution Models on Viroid and RNA Virus Phylogenies

**DOI:** 10.1093/gbe/evx273

**Published:** 2018-01-09

**Authors:** Juan Ángel Patiño-Galindo, Fernando González-Candelas, Oliver G Pybus

**Affiliations:** 1 Unidad Mixta Infección y Salud Pública FISABIO-Salud Púbica/Universitat de València-I2SysBio, València, Spain; 2 CIBER Epidemiología y Salud Pública, València, Spain; 3 Department of Zoology, University of Oxford, United Kingdom

**Keywords:** RNA virus, viroid, RNA secondary structure, phylogenetics

## Abstract

Many viroids and RNA viruses have genomes that exhibit secondary structure, with paired nucleotides forming stems and loops. Such structures violate a key assumption of most methods of phylogenetic reconstruction, that sequence change is independent among sites. However, phylogenetic analyses of these transmissible agents rarely use evolutionary models that account for RNA secondary structure. Here, we assess the effect of using RNA-specific nucleotide substitution models on the phylogenetic inference of viroids and RNA viruses. We obtained data sets comprising full-genome nucleotide sequences from six viroid and ten single-stranded RNA virus species. For each alignment, we inferred consensus RNA secondary structures, then evaluated different DNA and RNA substitution models. We used model selection to choose the best-fitting model and evaluate estimated Bayesian phylogenies. Further, for each data set we generated and compared Robinson–Foulds (RF) statistics in order to test whether the distributions of trees generated under alternative models are notably different to each other. In all alignments, the best-fitting model was one that considers RNA secondary structure: RNA models that allow a nonzero rate of double substitution (RNA16A and RNA16C) fitted best for both viral and viroid data sets. In 14 of 16 data sets, the use of an RNA-specific model led to significantly longer tree lengths, but only in three cases did it have a significant effect on RFs. In conclusion, using RNA model when undertaking phylogenetic inference of viroids and RNA viruses can provide a better model fit than standard approaches and model choice can significantly affect branch length estimates.

## Introduction

Many tasks in modern molecular systematics rely upon the use of nucleotide (or codon or amino acid) substitution models. Substitution models facilitate the statistical testing of molecular evolutionary hypotheses and improve the estimation of genetic distances among taxa by accounting for unobserved evolutionary changes. However, these models make several assumptions about the process of molecular evolution, for example, whether nucleotides differ in relative frequency, or whether substitution rates vary among nucleotides (Posada and Crandall, 2001) or codon positions (Shapiro et al., 2006).

The existence of RNA secondary structure, such as stems (also called hairpins), is likely to violate a key assumption of most methods of phylogenetic reconstruction, that evolutionary changes occur independently among sites (Nasrallah et al., 2011). Stems are comprised of nucleotide sequences that form base-pairings with complementary regions within the same strand. Among the 16 possible base-pairings that can potentially occur, only six (the Watson–Crick pairs AU, UA, GC, CG, and the “wobble” pairs GU and UG) are stable enough to form actual base-pairs (the remaining base-pairings are called mismatches, MM). RNA structures play important roles in many viruses and viroids, whose genomes are encoded in RNA. For example, RNA structures are involved in viral/viroid replication (Hutchins et al., 1986; Damgaard et al., 2004), translation (Pelletier and Sonenberg, 1988), and immune evasion (Tellam et al., 2008). Nucleotide changes that disrupt the most stable Watson–Crick pairs are often deleterious, and therefore, RNA secondary structures can impose strong evolutionary constraints on sequence evolution. In order to maintain RNA structure, a base of a pair must in many cases be matched by a complementary nucleotide. One consequence of this evolutionary constraint is that the amount of nucleotide evolution estimated from unpaired sites is expected to be higher than that from paired sites (Nasrallah et al., 2011). An association between the presence of complementary base pairing and amino acid conservation has been reported for HIV-1 (Sanjuán and Bordería, 2011; Snoeck et al., 2011).

In order to accommodate the evolutionary correlations among-sites that are imposed by RNA secondary structure, various types of RNA-specific substitution models for phylogenetic inference have been developed. The 6-state (RNA6A-E) models discard all mismatched sites from analysis, whereas the 7-state (RNA7A-G) models group all mismatched sites into a single state (Tillier and Collins, 1998). The 16-state models (RNA16A-F, I-K) take into account all 16 possible pairs that the four nucleotides could form (Schöniger and Von Haeseler, 1994; Muse, 1995). RNA16 models can be classified in three different types: 1) “all pairs” models (RNA16A, B, I, J, and K), in which each of the 16 dinucleotides has its own equilibrium frequency; 2) “stable sets” models (RNA16D, E, and F), in which the equilibrium frequencies of mismatched pairs, Watson–Crick pairs, and wobble pairs, are different; and 3) “stable pairs” model (RNA16C), which can be considered to be an extension of an RNA7 model, in which the ten possible mismatched pairs have a single equilibrium frequency (Savill et al., 2001; Allen and Whelan, 2014).

Previous studies of ribosomal RNA (rRNA) genes have concluded that RNA-specific models outperform standard nucleotide substitution models when describing the evolution of structured RNA sequences (Savill et al., 2001; Kosakovsky Pond et al., 2007), as assessed by statistical model comparison using the Akaike Information Criterion (AIC) (Linhart and Zucchini, 1986). The use of RNA models in rRNA phylogenetic inference has been associated with an improvement in accuracy (the distance between the real and the reconstructed tree) and robustness (as measured by bootstrap support values) (Keller et al., 2010). In agreement with these studies, Allen and Whelan (2014) compared different nucleotide and RNA models for 287 human RNA gene families, most of them microRNAs and snoRNAs, and concluded that RNA models outperformed nucleotide substitution models in most cases, because the former yielded the lowest corrected AIC (AICc) values.

Conserved RNA secondary structures have been reported to exist in the genomes of many linear RNA viruses, for example, species of the *Flaviviridae* family (Thurner et al., 2004; Mauger et al., 2015) and HIV-1 (Watts et al., 2009). Hepatitis Delta Virus (HDV) and viroids, which exist as circular RNA genomes, present exceptionally highly structured genomes and >70% of the nucleotide sites in their genomes form base-pairs (Wang et al., 1986; Sanjuán et al., 2006). Despite this, phylogenetic reconstructions of RNA viruses (including HDV) and viroids have not been generated using RNA models, and thus potentially ignore the constraints that these structures impose on genome evolution.

The goal of this study is to investigate whether RNA-specific substitution models outperform standard nucleotide substitution models when applied to different sets of full-genome sequences from RNA viruses and viroids. Further, we measure the degree to which phylogenetic inference is affected, in terms of estimated branch lengths and tree topologies, when an RNA-specific model is used to describe the evolution of paired sites in the genomes of these infectious agents.

## Materials and Methods

### Data Sets and Alignments

Full-genome nucleotide sequences from six viroid species [tomato apical stunt pospiviroid (TASVd), citrus exocortis viroid (CEVd), columnea latent viroid (CLVd), grapevine yellow speckle viroid (GYSVd), Australian grapevine viroid (AGVd), potato spindle tuber viroid (PSTVd)], and ten single-stranded RNA virus species [hepatitis delta virus (HDV), Sudan-ebolavirus (SUDV), dengue virus (DENV), hepatitis C virus (HCV), human immunodeficiency virus (HIV), foot and mouth disease virus (FMDV), measles virus (MeV), rabies virus (RV) rubella virus (RuV), and mumps virus (MuV)] were downloaded in April, 2015. Viroid and HDV sequences were downloaded from GenBank; viral genomes were obtained from the Virus Pathogen Database and Analysis Resource, VIPRBRC (http://www.viprbrc.org). Only full genome sequences that included untranslated regions were considered. Alignments for each species were generated using MAFFT (using the “align- G-ins- 1” progressive method strategy) (Katoh and Standley, 2013) and positions with a high proportion of gaps were removed with TrimAl (Capella-Gutiérrez et al., 2009). Given that “gappy” positions were rare and represented rare insertions that were absent in most taxa, excluding them had no influence on the inferred consensus RNA secondary structures for each species.

### RNA-Secondary Structure Inference

For each species, RNA minimum free-energy (MFE) consensus secondary structures were predicted using RNAalifold, as implemented in the Vienna Package 2.0 (Lorenz et al., 2011). The folding temperature was set to 25 and 37 °C for viroids and viruses, respectively, which, according to Sanjuán et al. (2006), corresponds to the temperatures at which these pathogens replicate. RNA molecules were assumed to be circular for HDV and viroids. Because the large size of RNA viruses with linear genomes (at least 8,000 nt) can hinder the inference of RNA secondary structure, the RNAalifold analyses of these data sets were performed using segments of 1,000 nt. Analyses of HIV and HCV were also performed using the RNA structures obtained experimentally using approaches based on SHAPE reactivity, as reported by Siegfried et al. (2014) and Mauger et al.(2015), respectively. Arc diagrams of the obtained structures, which display the locations of base-paired nucleotides along each genome, were plotted with the R4RNA package for R (Lai et al., 2012; R core team, 2015).

The conservation of RNA secondary structure within each data set was tested using RNAz (Gruber et al., 2007) by calculating the Structure Conservation Index (SCI). An SCI = 0 indicates that RNAalifold did not find a consensus structure, whereas a SCI ≈ 1 reflects a set of perfectly conserved structures (Washietl et al., 2005). Consequently, only those data sets with an overall SCI ≥ 0.70 were retained for further analysis, in order to ensure that the RNA secondary structures under investigation were evolutionary conserved.

In order to assess the order-dependency of the inferred RNA secondary structures, a sequence randomization method (Simmonds et al., 2004; Davis et al., 2008) implemented in the SSE 1.1 package (Simmonds, 2012) was applied to each data set. This method evaluates the difference between the MFE of the inferred secondary structure from 1) real sequences from each alignment and 2) the same sequences after their sites have been randomly reordered. The sequence randomization is undertaken in a manner that preserves dinucleotide frequencies. For viroids and HDV, these MFE differences (MFED) were calculated in windows of size 300 nt, and a sliding-step of 30 nt, under the constraint of a circular genome. For the RNA viruses with linear genomes, MFED were calculated for each 1,000-nt long segment. In all cases, MFEDs were calculated under both sense and antisense orientations. A positive MFED indicates that the MFE of the RNA structure derived from the real sequence alignment is lower (and thus more stable) than that from the randomized sequence alignment, and thus is a conservative test of the presence of a significantly structured genome.

### Model Selection and Phylogenetic Analyses

For each data set, the best-fitting substitution model for phylogenetic reconstruction was chosen using a Perl script included in the package PHASE-3.0 (“model_selection.pl”; Allen and Whelan, 2014). The inputs to this analysis were 1) the sequence alignment, 2) the inferred secondary structure, and 3) an initial neighbor-joining tree, estimated under the Tamura-Nei model, using Mega version 5 (Tamura et al., 2011). The Perl script compares an array of different models: two DNA substitution models (HKY and GTR), 16 different RNA substitution models (seven RNA7 and nine RNA16 models), and the inclusion or exclusion of a gamma distribution model of among-site rate variation. The best-fitting model was identified as that with the lowest value of the corrected Akaike Information Criterion (Akaike, 1974; Burnham and Anderson, 2002): AICc= −ln(*L*) + 2*k* + 2*k*(*k* + 1)/(*n* − *k* − 1), where *k* is the number of parameters, *L* is the likelihood, and *n* is sample size.

Phylogenetic trees were estimated using the Bayesian Monte Carlo Markov Chain (MCMC) approach implemented in the program mcmcphase, which is part of the package PHASE-3.0. This program allows the inference of a phylogenetic tree under a “mixed model,” in which a DNA substitution model is assigned to unpaired positions and an RNA substitution model is assigned to paired positions. For each data set, two different phylogenetic trees were estimated, using either 1) the best-fitting model (which in our study was always a mixed model) or 2) a DNA-only model. At least two independent MCMC runs, each with >1,000,000 states, were computed, and a 10% burn-in was removed from each run before analysis. The prior distribution used for branch length estimation was an exponential distribution with rate parameter = 10. This is the default prior in PHASE-3.0.

After combining the output of both MCMC runs, convergence was checked visually by plotting sampled values of the likelihood, posterior and priors. After convergence was confirmed, an extended majority rule consensus phylogenetic tree was obtained for each data set using the program “mcmcsummarize” from the PHASE package. The phylogeny obtained under the best-fitting model (which, for all the data sets, was the mixed model) was then used as a fixed topology to estimate branch lengths, by running mcmcphase with either the DNA or the mixed substitution model.

Next, sites in each sequence alignment were partitioned into two separate data sets that included only paired or unpaired sites, respectively. Branch lengths were estimated separately from these two partitions, using the same fixed topology as above. A DNA substitution model was used for the unpaired sites partition, and either the best-fitting DNA substitution model or the RNA substitution model was used for the paired sites partition.

### Comparison of Branch Lengths and Tree Topologies

Tree lengths (the sum of all branch lengths in a phylogeny) were calculated from the consensus trees that were estimated from the complete alignments. Tree lengths obtained from paired sites (either under a DNA or RNA substitution model) and unpaired sites (always under a DNA substitution model) were calculated in the same way. To determine if branch lengths estimated under the DNA and mixed substitution models were different, they were compared using paired Wilcoxon tests.

To assess the effects of model choice on inferred tree topologies, we computed distributions of Robinson–Foulds (RF) distances. The RF distance between two tree topologies is a measure of how different they are (Robinson and Foulds, 1981). For each data set we computed three different distributions of RF distances: 1) distances between pairs of topologies that were sampled from the same posterior distribution, generated using a RNA-specific substitution model, 2) distances between pairs of topologies sampled from the same posterior distribution, generated using a standard DNA substitution model, and 3) distances between a tree from the posterior used in (1) and a tree from the posterior used in (2). In total, 18,000 trees were sampled from each posterior distribution. For cases (1) and (2), trees were sampled without replacement, to prevent MCMC states being compared with themselves. All RF distances from a given data set were normalized according to the number of taxa (by dividing the RF value by 2·*n*-6, where *n* is the number of taxa to be analyzed). Distributions (1) and (2) represent the degree of statistical uncertainty in tree topologies arising from inference under a given substitution model, whereas distribution (3) represents the difference in tree topologies obtained by inference under the two different models. Thus, a comparison of distribution (3) with distributions (1) and (2) indicates whether the effect on tree topology of using an RNA-substitution model is greater or less than estimation uncertainty alone.

We assessed whether distributions (1) and (22) were significantly different from distribution (3) by performing 9,000 pairwise comparisons between RF distances randomly sampled from distributions (1) or (2) and from distribution (3). The probability that the two distributions are different is computed as the number of instances in which the RF distance from (3) is larger than that from (1) or (2), divided by the total number of comparisons (Abecasis et al., 2009). P-values obtained from the same virus/viroid were then corrected with the false discovery rate method (FDR; Benjamini and Hochberg, 1995). The distributions of normalized RF distances and their statistical comparisons were computed using an R script (available from https://github.com/juanangel87/GBE_2017) that utilizes the phangorn package for R (Schliep, 2011).

In order to assess whether the joint prior was having undue influence over the estimated posterior distributions for branch lengths and RF distances, we computed one of the data sets (HCV-1b) without data, such that the MCMC sampled from the prior distribution only, for all the models implemented in PHASE-3.0 (GTR, HKY, RNA6A-E, RNA7A-G, and RNA16A-F, I-K). Using the comparison approach described above, we then compared the branch lengths and tree topologies inferred from the HCV-1b data set (under the GTR and RNA16A models) to those obtained without data.

## Results

### RNA Secondary Structure Inference

Structure Conservation Index (SCI) values were calculated for each data set. Values of SCI ≤ 0.70 were found in only five viral data sets: HCV (SCI = 0.40), DENV (0.40), HIV-1 (0.66), RV (0.66), and HDV (0.66). These data sets include five of the seven data sets with the largest average pairwise genetic distances (table 1). For genetically diverse viruses such as these, evolutionary conservation of RNA secondary structure will be greater at the sub-genomic level. Therefore, for DENV, HIV-1, RV, and HDV we attempted to infer RNA secondary structures for taxonomic units below the species level (i.e., subtypes, genotypes, etc.). For HDV, we found 46% of paired sites along the genome were conserved among the eight HDV genotypes in the virus, each with SCI > 0.70 separately. For HCV, HIV-1, DENV, and RV, it was necessary to analyze a less diverse sub-genomic taxonomic unit (specifically, subtype 1 b for HCV, genotype 1 for DENV, subtype B for HIV-1, and lineage C1 for RV). All these genotype/subtype data sets had SCI > 0.70 and were therefore analyzed further. Arc diagrams representing the RNA minimum free-energy consensus secondary structures obtained with RNAalifold for each data set with SCI > 0.70 are shown in supplementary figure S1, Supplementary Material online. The percentage of nucleotides forming base-pairs in the alignments that were further analyzed ranged between 23% (HIV, structure obtained experimentally using SHAPE by Siegfried et al., 2014) and 78% (AGVd) (table 1).

**Table 1:** Summary Statistics of Each Viroid and Virus Data Set Analyzed, Including Size (number of taxa and sequence length), Overall Mean Genetic Distance, Structure Conservation Index (SCI), Estimate Percentage of Base-Paired Nucleotides, Median MFED Value, and Best-Fitting Evolutionary Model of Each Viroid and Virus Data Set Analyzed

	*n* Taxa	Sequence Length (nt)	Mean *P* Distance	SCI	% (paired nucleotides)	%(Median MFED)	Best-Fitting Model	Δ AICc (overall best-fitting model vs. best-fitting DNA-only model)
Viroids								
TASVd	22	374	0.036	0.91	68	14.30	HKY_Γ+RNA16C_Γ	338
CeVd	178	369	0.041	0.92	70	8.40	GTR_Γ+RNA16E_Γ	258
CLVd	14	379	0.061	0.88	68	15.40	GTR_Γ+RNA16A_Γ	352
GYSVd	24	352	0.128	0.84	65	8.30	GTR_Γ+RNA16C_Γ	336
AGVd	27	368	0.02	0.91	78	11.40	HKY_Γ+RNA16C	295
PSTVd	88	356	0.019	0.97	69	12.80	HKY_Γ+RNA16A_Γ	220
Viruses								
HDV	121	1,543	0.204	0.66^a^	46^b^	2.60	GTR_Γ+RNA16D_Γ	2,237
Sudan Ebolavirus	7	18,875	0.032	0.9	64	1.40	GTR_Γ+RNA16A	>1,000
DENV	23	10,733	0.263	0.40^a^	NC	NC	NC	NC
DENV-1	20	10,733	0.061	0.81	60	(−)1.3	GTR_Γ+RNA16A_Γ	>1,000
HCV	42	9,605	0.292	0.40^a^	NC	NC	NC	—
HCV-1b (RNAalifold)	20	9,605	0.087	0.82	66	3.80	GTR_Γ+RNA16A_Γ	>1,000
HCV-1b (SHAPE reactivity)	20	9,605	0.087	0.82	51	3.80	GTR_Γ+RNA16A_Γ	>1,000
HIV-1	18	9,173	0.126	0.64^a^	NC	NC	NC	—
HIV-1B (RNAalifold)	33	9,173	0.056	0.74	57	0.50	GTR_Γ+RNA16D_Γ	>1,000
HIV-1B (SHAPE reactivity)^c^	33	9,173	0.056	0.74	23	0.50	GTR_Γ+RNA16D_Γ	674
FMDV	19	8,192	0.135	0.75	60	3.90	GTR_Γ+RNA16D_Γ	>1,000
Measles	20	15,893	0.042	0.89	63	0.10	GTR_Γ+RNA16A_Γ	>1,000
Rubella	35	9,758	0.06	0.9	65	1.20	GTR_Γ+RNA16A_Γ	>1,000
Mumps	20	15,355	0.045	0.86	61	(−)0.8	GTR_Γ+RNA16A_Γ	>1,000
Rabies	26	11,923	0.111	0.66	5^b^	NC	NC	NC
Rabies C1	20	11,923	0.088	0.74	63%	(−)0.3	GTR_Γ+RNA16A_Γ	>1,000

Note.—NC, not computed.

a SCI (Structure Conservation Index) below 0.70.

b Percentage of nucleotides forming base pairing, after obtaining a consensus structure comprising paired-sites that are present in >75% of genotypes/subtypes within a species.

c The RNA secondary structure only includes the 15 regions along the HIV-1B genome, reported by Siegfried et al. (2014), that have both SHAPE reactivity values and low Shannon entropies, thus being considered as well defined structures.

The median MFED values we obtained for viroids and HDV ranged between 2.6% (HDV) and 15.4% (CLVd) and, in almost all cases, were higher than those obtained for viruses with linear RNA genomes. Only FMDV and HCV-1 b presented median MFED values higher than 2%; in most viruses this value was close to zero (table 1).

### Model Selection and Phylogenetic Analyses

For each data set analyzed, the best-fitting model (i.e., the model with the lowest AICc value) was a mixed model, which assigned a DNA substitution model (either GTR or HKY) to unpaired sites and a RNA16 substitution model to paired sites (table 1).

Phylogenies were estimated for each data set using mcmcphase (part of the PHASE-3.0 package). To examine the effect of including a RNA substitution model in the analysis, we estimated branch lengths on a fixed topology under two different substitution models: first, using the best-fit model (which, as noted above, was always a mixed model), and second, using the best-fitting DNA substitution model. Tree lengths (the sum of all branch lengths) obtained under the two abovementioned models (termed *L*(mixed) and *L*(DNA)) were compared using paired Wilcoxon tests. We also calculated ratios of the tree lengths obtained under the two models (i.e., *L*(mixed)/*L*(DNA)) (see table 2). Although the effect on branch length estimates of using a mixed model was near to zero for PSTVd and AGvd (ratios = 0.99 and 1.00, respectively; *P* values > 0.05), for the other viral and viroid data sets there was a significant increase in tree length (*P* values < 0.05). The largest effects were observed for TasVd, CLVd, and GYSVd, whose *L*(mixed)/*L*(DNA) ratios were 6.5, 2.8, and 2.7, respectively.

**Table 2:** Comparisons of Tree Lengths (*L*) Estimated under DNA and Mixed Models, for All Sites, Paired Sites, and Unpaired Sites

	*L* (DNA model)	*L* (mixed model)	Ratio (mixed/DNA)	*P* value log(DNA vs. mixed)^a^	*L* (unpaired sites)	*L* (paired sites, DNA model)	*L* (paired sites, RNA model)	Ratio (paired-DNA model/unpaired)	Ratio (paired-RNA model/unpaired)
Viroids									
TASVd	0.47	3.07	6.532	<0.001	3.05	0.51	1.82	0.167	0.597
AGVd	4.91	4.93	1.004	0.341	0.55	0.31	1.13	0.563	2.055
CeVd	30.41	33.81	1.111	<0.001^c^	34.18	33.4	31.42	0.977	0.919
CLVd	0.44	1.23	2.795	<0.001	1.36	0.74	1.01	0.544	0.743
GYSVd	0.77	2.06	2.675	<0.001	2.11	0.88	2.21	0.417	1.047
PSTVd	17.28	17.05	0.989	0.14	17.22	17.17	17.13	0.997	0.995
Viruses									
HDV	9.09	12.15	1.337	<0.001	12.44	7.5	15.35	0.603	1.234
Sudan Ebolavirus	0.07	0.1	1.408	<0.001	0.1	0.05	0.13	0.555	1.322
DENV-1	0.46	0.55	1.196	<0.001	0.55	0.42	0.92	0.764	1.673
HCV-1b (RNAalifold)	1.16	1.73	1.495	<0.001	1.73	0.89	1.84	0.513	1.064
HCV-1b (SHAPE)	1.17	1.4	1.191	<0.001	1.4	0.98	1.9	0.7	1.357
HIV-1B (RNAalifold)	1.48	2.21	1.493	<0.001	2.21	0.92	2.2	0.416	0.995
HIV-1B (SHAPE)^b^	1.48	1.51	1.02	<0.001	1.52	1.5	2.59	0.987	1.704
FMDV	2.01	2.48	1.234	<0.001	2.52	1.48	2.72	0.587	1.079
Measles	0.33	0.42	1.273	<0.001	0.42	0.3	0.78	0.714	1.857
Rubella	0.71	1.04	1.465	<0.001	1.04	0.6	1.33	0.577	1.277
Mumps	0.35	0.48	1.371	<0.001	0.42	0.32	0.86	0.761	2.048
Rabies C1	0.94	1.16	1.234	<0.001	1.16	0.86	2.07	0.741	1.784

a *P* value obtained from comparing the branch length distributions using paired Wilcocon tests, after a logarithm transformation.

b The RNA secondary structure only includes the 15 regions along the HIV-1B genome, reported by Siegfried et al. (2014), that have both SHAPE reactivity values and low Shannon entropies, thus being considered as well defined structures.

c Topology could not be fixed for branch lengths inference due to unresolved bipartitions, and a Wilcoxon rank sum test was performed instead of a paired test.

We also compared the estimated tree lengths obtained from the separate data sets comprising unpaired and paired sites. The *L*(paired)/*L*(unpaired) ratios obtained under the DNA model reported in table 2 are consistent with the hypothesis that base-pairing imposes a significant evolutionary constraint. With the exception of CeVd, PSTVd, and HIV-1B (SHAPE) tree lengths estimated from paired sites were >29% shorter than those estimated from unpaired sites. However, when an RNA model was used for paired sites, the *L*(paired)/*L*(unpaired) ratios increased and, in most cases, paired sites under an RNA model yielded remarkably larger tree lengths than unpaired sites (AGVd, HDV, MeV, SUDV, DENV-GT1, HCV-1b-SHAPE-, HIV-1B-SHAPE-, FMDV, MeV, RuV, MuV, RV) (table 2).

**Fig. 1.**
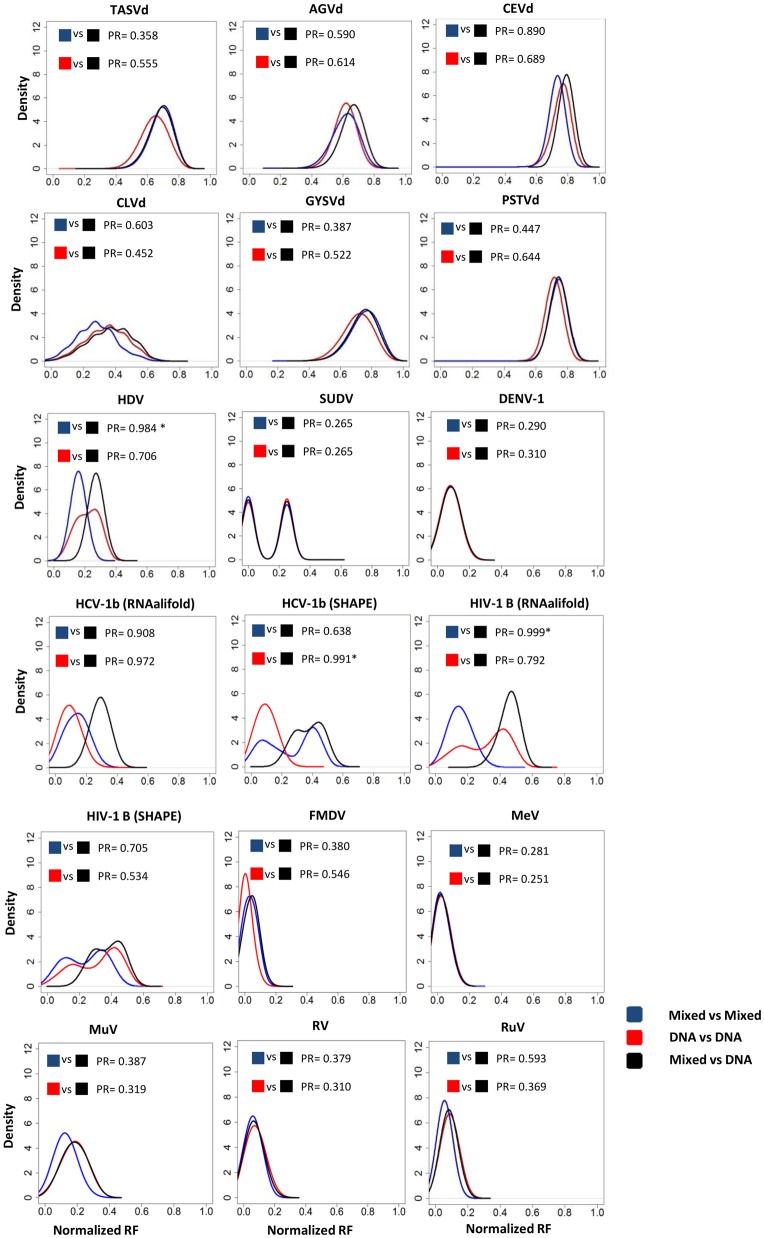
—Density plots representing, for each data set, the distribution of RF distances obtained by comparing topologies from the same posterior distribution (either including or excluding the RNA model) versus the distribution of RF distances obtained by comparing topologies from two different posterior distributions. The results of the randomization tests are shown as the proportion of comparisons for which an RF distance obtained through comparing states from the same posterior (blue = under mixed model; red = under DNA model) was lower than the RF distance obtained by comparing states from the two different posterior distributions (black= mixed vs. DNA models). Significant values after FDR correction are labeled with “*.”

For each data set analyzed, three different RF distance distributions were obtained as described above. The results are shown in figure 1. The randomization tests showed that, after FDR correction of *P* values, only for HDV, HCV-1b (SHAPE), and HIV-1B (RNAalifold) did we obtain a significantly different distribution of RF distances when comparing topologies sampled from the same posterior than when comparing topologies from the two different posterior distributions. For both HDV and HIV-1B we observed shorter RF distances under the mixed model (HDV: *P* value = 0.016; HIV-1B: *P* value= 0.002). For HCV, shorter RF distances were obtained when comparing topologies sampled under the DNA model with those obtained by comparing different posterior distributions (*P* value = 0.018). For HDV, the consensus phylogenetic tree obtained under the mixed model presented more highly supported nodes (defined by posterior node probabilities ≥ 0.90) than those obtained under a DNA-only model: 82 (mixed model) versus 68 (DNA model). The same effect was observed in HIV-1B (RNAalifold): 21 well supported nodes (mixed model) versus 15 (DNA model), but the differences were reduced when using the SHAPE-derived secondary structure (17 well-supported nodes using the mixed model, and 15 using the DNA model). In the case of HCV-1b, using the secondary structure derived from RNAalifold had no effect on the number of well-supported clades (14 under both models). However, the use of the experimentally derived structure led to a lower number of well-supported clades (from 14, using the DNA model, to 10, using the mixed model) (supplementary fig. S2, Supplementary Material online). The RF distances obtained when comparing the consensus trees (DNA vs. mixed model) of these data sets were 0.22 for HDV, 0.24 for HCV-1 b (RNAalifold), 0.35 for HCV-1 b (SHAPE), 0.37 for HIV-1B (RNAalifold), and 0.33 for HIV-1B (SHAPE).

For all data sets, branch lengths obtained by sampling only from the joint prior distribution were significantly longer than those obtained by sampling from the data-informed marginal posterior distribution with empirical data (all *P* values < 0.001) (supplementary table S1, Supplementary Material online). Similarly, RF distributions from the marginal posterior were significantly shorter than those obtained by sampling only from the prior (all *P* values < 0.001) (supplementary fig. S3, Supplementary Material online). Thus, under the different models implemented in PHASE-3.0, the empirical data are informative and the joint prior appears to have limited influence on the estimated posterior distributions.

## Discussion

We assessed the effect of RNA substitution models on the inference of genetic distances and phylogenies for viroids and RNA viruses using complete genome sequences. We first investigated whether using an RNA-specific model provides a better fit to the data than the conventional DNA substitution models that are widely used to study viral evolution. In all data sets the best-fit model was a mixed model that uses a nucleotide model for unpaired sites and a RNA model for paired sites. These mixed DNA/RNA models outperformed models in which unpaired and paired sites were partitioned and represented by different DNA models. It is important to note that 16-state RNA substitution models outperformed 7-state RNA models in all instances. The main difference between these families of RNA models is that 7-state models pool all mismatches (pairs of nucleotides that do not form stable base pairs) in a single state while 16-state models consider each mismatch as separate state. A special case is RNA16C, in which the ten different mismatched pairs have the same transition probabilities, and is thus considered an extension of an RNA7 model (Savill et al., 2001).

For most of the viroids we studied, the RNA16C model was the best-fitting model, whereas for the RNA viruses, RNA16A was the best-fitting model in most cases. RNA16A and RNA16C have been reported previously to fit well when applied to noncoding RNA data sets because, unlike other RNA16 models, they allow a nonzero rate of double substitutions, and thus they count complementary changes as a single step (Savill et al., 2001). Allen and Whelan (2014) assessed best-fitting models for the analysis of the evolution of human noncoding RNAs and found that, for the majority of RNA types, “stable pairs” models (RNA7A-G and RNA16C) and “stable sets” models (RNA16D, E, F) fitted the best for such data. They concluded that the former were usually selected when applied to data sets in which few evolutionary changes occurred, whereas the latter were selected when the consensus secondary structure contained higher proportions of paired sites. Our results suggest that models that allow for nonzero rates of double substitutions fit best for viroid and virus genome data sets.

Bayesian phylogenies were estimated using the best-fitting mixed model and using a DNA substitution model. This allowed us to assess the differences in estimates of branch lengths and trees topologies when an RNA model is included in the phylogenetic analysis. In all data sets (except PSTVd and AGVd) the use of a RNA model led to trees with substantially longer branch lengths. Among those data sets where the use of an RNA model led to a significant increase in branch lengths, the increase in total tree length ranged between 2% (HIV-1B, SHAPE structure) and 653% (TASVd). Under a DNA model, tree lengths estimated from paired sites were always much shorter than those estimated from unpaired sites, and such differences were reduced when the RNA model was applied to paired sites. A lower number of substitutions at paired sites, compared with unpaired sites, is expected due to the likely stronger evolutionary constraints at paired sites (Nasrallah et al., 2011). However, in some data sets tree lengths estimated from paired sites under a RNA model were considerably larger than those estimated from unpaired sites (especially in AGVd, measles, mumps, and rabies virus; table 2). These results suggest that, in such cases, RNA models may overestimate the number of substitutions along the inferred tree. It is important to note that PHASE-3.0 estimates branch lengths in units of expected number of substitutions per nucleotide, even when a RNA model is included (and not the number of substitutions per base-pair). We recognize the benefit of this parameterization, because it allows us to directly compare branch lengths estimated under different models (Allen and Whelan, 2014).

In our analysis, viroid phylogenies exhibited larger RF distances between trees sampled from posterior distributions than did the virus phylogenies, regardless of the evolutionary model used. This suggests a greater degree of uncertainty in estimated viroid phylogenies, possibly reflecting lower phylogenetic signal in viroid alignments. Furthermore, the comparisons of RF distributions show that, with the exception of HCV, HIV, and HDV, the use of a mixed model to infer viral and viroid phylogenies has no significant effect on estimated tree topologies. For HCV, HDV, and HIV-1, including an RNA model was associated with an increase in the number of well-supported branches in the resulting consensus tree.

The RF distance distributions for SUDV, HCV-1b (SHAPE), and HIV-1B (RNAalifold and SHAPE) were bimodal. For SUDV this is likely because there were comparatively few sequences, that is, RF distances were zero or very low because sampled tree topologies were identical or very similar. In the case of HIV-1B (RNAalifold), the RF distance distribution obtained from the posterior distribution generated under the RNA model was less bimodal than the other distributions. In this case, the topological uncertainty under the mixed model was substantially reduced, as also reflected in improved node support values. In the consensus tree, the number of well-supported branches increased from 15 (DNA-only model) to 21 (mixed model). This, however, did not occur when using the RNA structure obtained with SHAPE. Indeed, in the case of HCV-1b, the RF distance distribution obtained from the posterior distribution generated under the DNA-only GTR model was lower and less bimodal than the other distributions. Thus, although RNA models for phylogenetic inference have been previously associated with an increase in branch support values (Keller et al., 2010), using an RNA model may also lead to higher topological uncertainty for some data sets (e.g., HCV-1b in our study). This could be due to the higher number of parameters to be estimated in RNA models.

One of the limitations that may hamper the use of RNA models for phylogenetic inference is the lack of reliable and representative RNA structures at the taxonomic unit under investigation. In this study, we used consensus RNA structures inferred by computational approaches. The accuracy of these RNA structures used could in theory be improved by using experimental approaches, such as RNAse mapping or SHAPE reactivity (Wilkinson et al., 2006). Although the bioinformatic tools used in this study (specifically RNAz and RNAalifold) showed the presence of ample conserved RNA secondary structures in the genomes analyzed, subsequent analyses that compared MFEs between true and randomized sequences suggest weaker support for some of these structures, at least for linear RNA viruses. However, the randomization test may be statistically conservative and further experimental analyses are needed. Indeed, although HCV-1b and HIV-1B presented negative MFED values, well defined and large scale RNA secondary structures for these viruses have been identified experimentally (Watts et al., 2009; Siegfried et al., 2014; Lavenderet al., 2015; Mauger et al., 2015). However, further analyses are necessary to assess the biological importance of such experimentally found structures.

To date very few secondary structures of complete viral genomes have been obtained experimentally and, further, they have been obtained from single genome sequences and thus do not capture the diversity in RNA secondary structures that is known to exist, even below the species level (Tuplin et al., 2004; Mauger et al., 2015). Because of this lack of representative experimental RNA secondary structures, we used the computational method implemented in RNAalifold, which allowed us to infer a consensus structure from alignments of different, yet related, RNA sequences. This method can improve the prediction of secondary structures compared with those obtained only with individual sequences, and can provide a representative structure for the analyzed data set (Hofacker et al., 2002; Bernhart et al., 2008). Furthermore, in our analyses we only included those data sets that represented taxonomic levels showing evolutionarily conserved structures, in order to ensure that the structures we inferred fitted well for each data set. However, it is important to note that, in vivo, the same primary sequence can fold into alternative structures (Schultes and Bartel, 2000). Consequently, differences between RNA structures in vivo and computationally inferred structures are expected to exist, and such differences are likely to be larger for viruses with linear RNA genomes than for HDV or viroids, which tend to form simpler, rod-like structures. For this reason, our in silico results should be interpreted with some caution, but will hopefully serve as a starting point for subsequent in vitro or in vivo research. For HIV-1B and HCV-1b we also undertook analyses using an experimentally determined RNA secondary structure; reassuringly, we obtained under both approaches similar results regarding best-fitting models and estimated branch lengths. Consequently, we recommend that RNA secondary structure is considered in phylogenetic inference only if the data set shows evidence of evolutionarily conserved structures. In addition, although the in silico prediction of consensus secondary structures for a given data set is preferable to the use of structures predicted from individual sequences, we recommend that in silico predicted structures are compared with those obtained from experimental analyses wherever possible.

In summary, we found that for all viroid and RNA virus data sets analyzed, the existence of RNA secondary structures can have significant effects on phylogenetic reconstructions. In all cases, assigning an RNA model to paired sites outperformed the use of a DNA-only model for phylogenetic reconstruction from virus and viroid complete genome sequences. Significant effects on phylogenetic branch lengths were also seen for most data sets. However, with a few exceptions, the use of an RNA-specific substitution model does not have a noticeable effect on the topology inferred. Further, the high statistical uncertainty that characterizes phylogenetic inference of viroid data sets did not decrease when RNA-specific models were used.

Previous phylogenetic analyses of viral and viroid genomes have been undertaken using DNA substitution models. However, in the light of our results, we suggest that such analyses should consider the inclusion of RNA models, as they may better describe the evolution of paired sites. In addition, it would be valuable if phylogeny software that implements molecular clock models, such as BEAST (Drummond and Rambaut, 2007), also includes the option of using RNA substitution models, as diversification dates and evolutionary rates inferred for RNA viruses under RNA models may be different from those obtained without considering RNA secondary structure. Resolving this question is an important topic for future research.

## Supplementary Material

Supplementary data are available at *Genome Biology and Evolution* online.

## Supplementary Material

Supplementary Figures
