# Supplementary material for: The Effect of RNA Substitution Models on Viroid and RNA Virus Phylogenies
Source: Genome Biol Evol. 2018 Jan 9;10(2):657–66. doi: 10.1093/gbe/evx273 (PMC5814974; doi:10.1093/gbe/evx273)

Supplementary Figure S.1

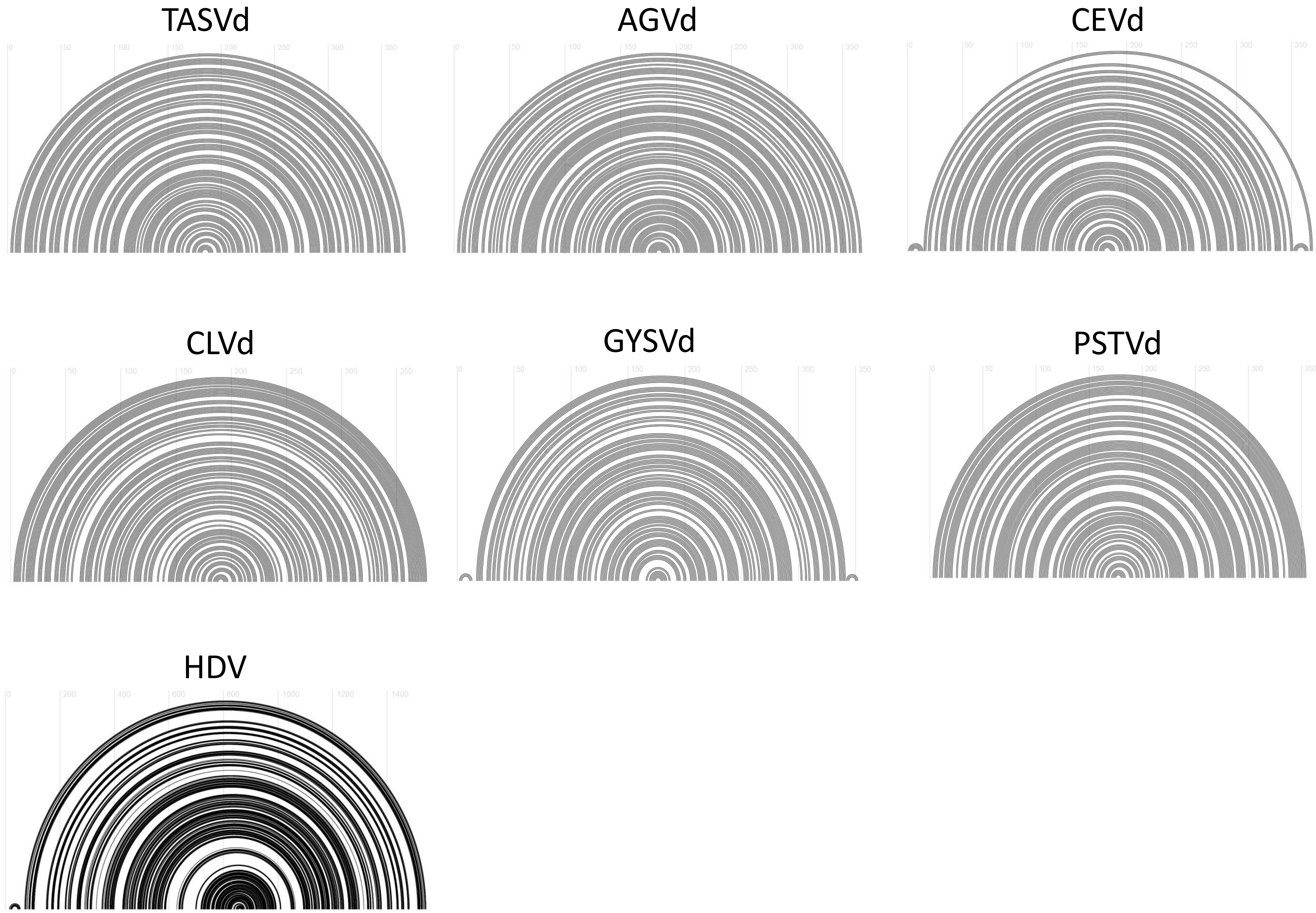

Supplementary Figure S.1 (cont)

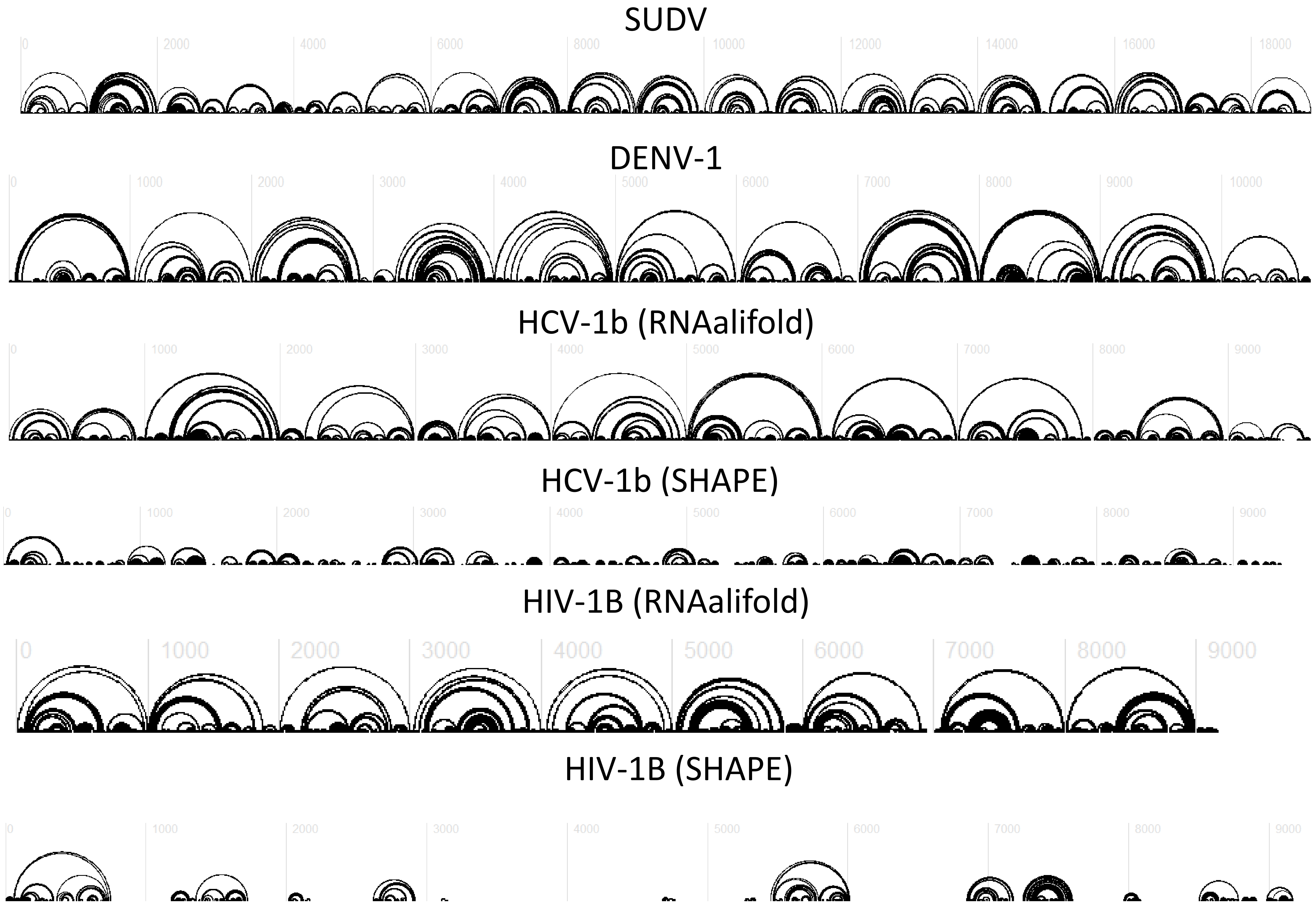

Supplementary Figure S.1 (cont)

FMDV

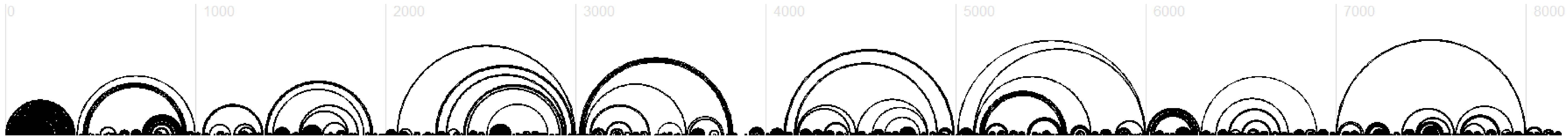

MeV

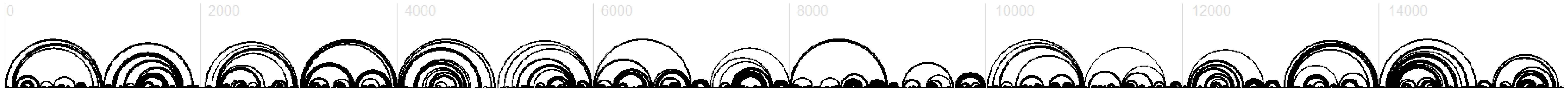

RuV

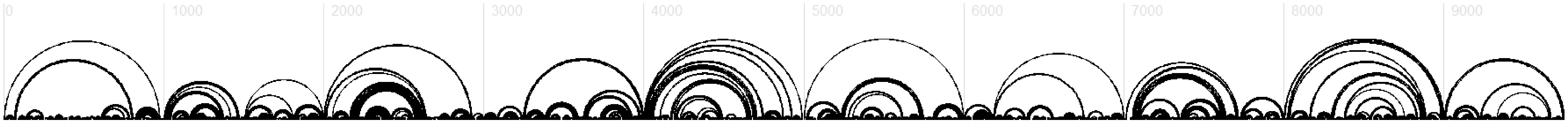

MuV

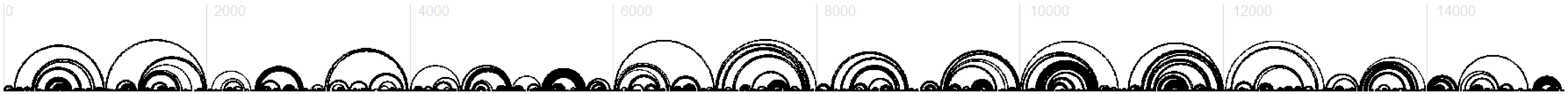

RV-C1

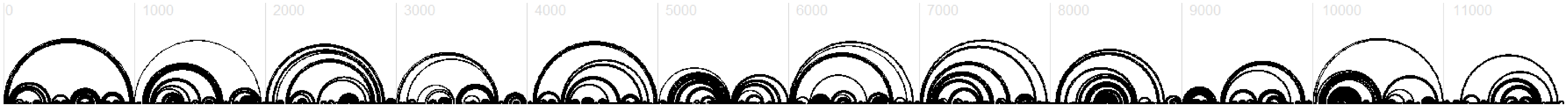

Supplement: Supplementary Figures [file evx273_supp.zip › SF1_STRsv2_2017.pdf]
