# Supplementary material for: The Effect of RNA Substitution Models on Viroid and RNA Virus Phylogenies
Source: Genome Biol Evol. 2018 Jan 9;10(2):657–66. doi: 10.1093/gbe/evx273 (PMC5814974; doi:10.1093/gbe/evx273)

Supplementary Figure S.2

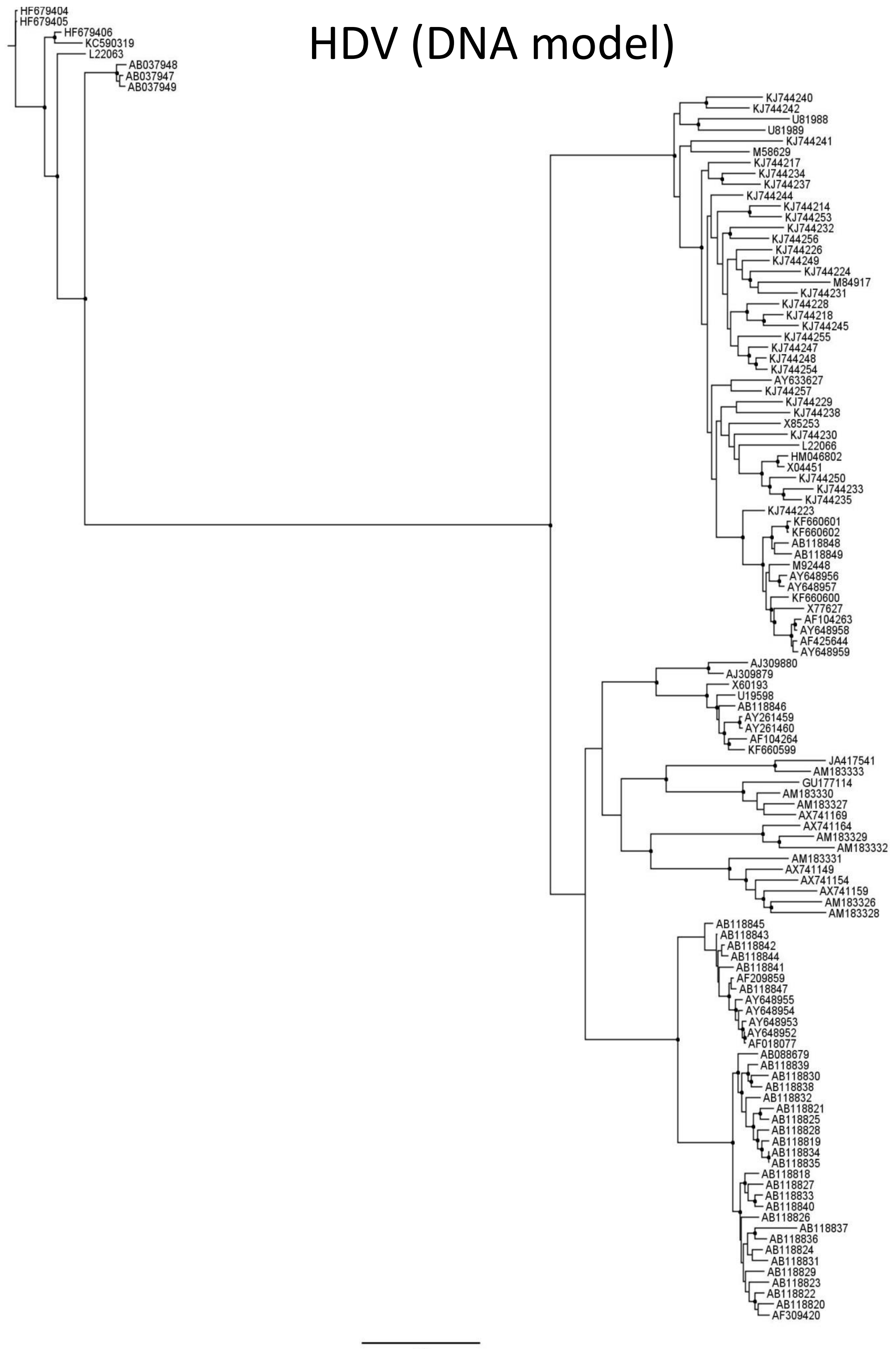

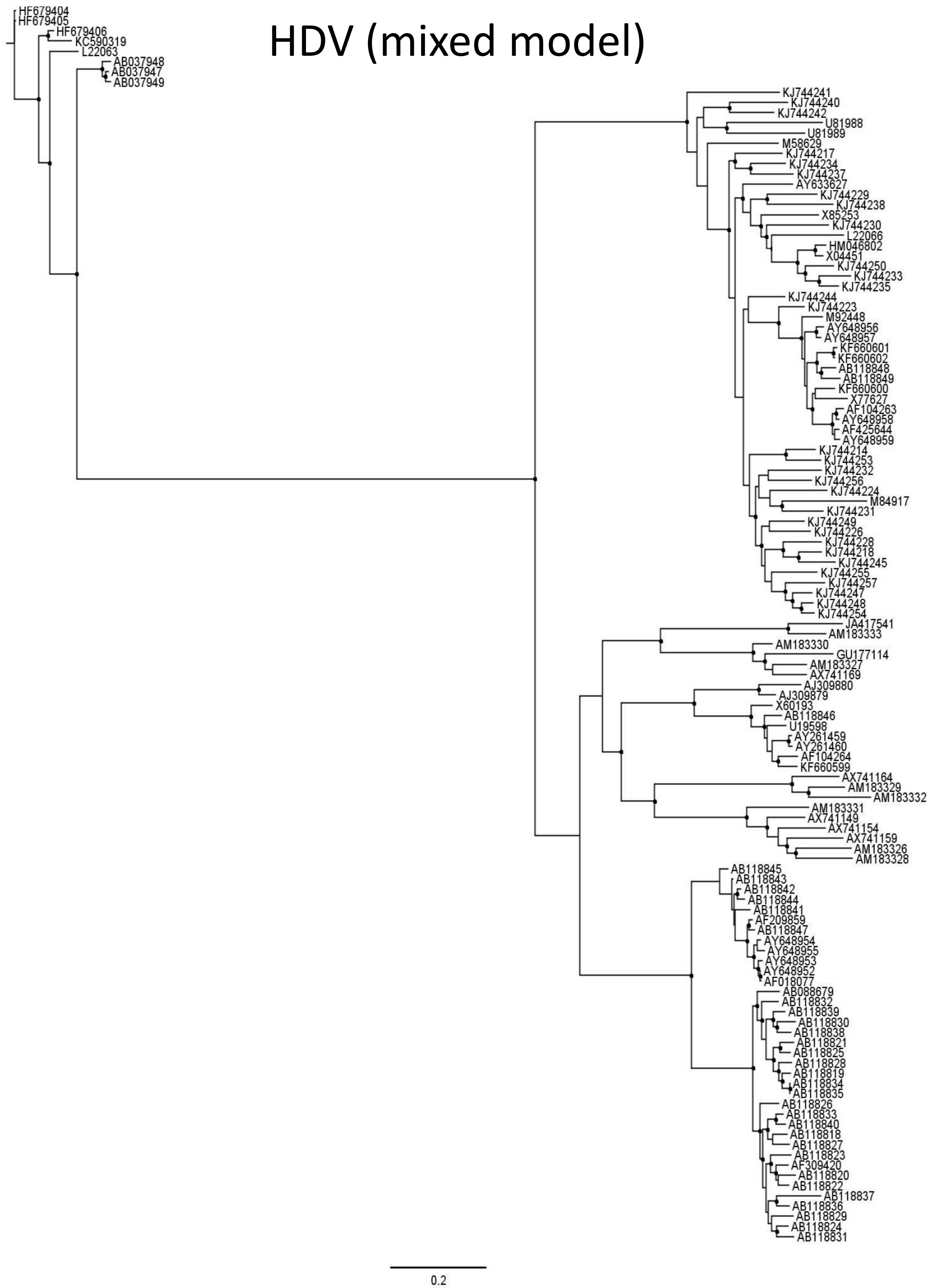

Supplementary Figure S.2 (cont)

HCV-1b RNAalifold (DNA model)

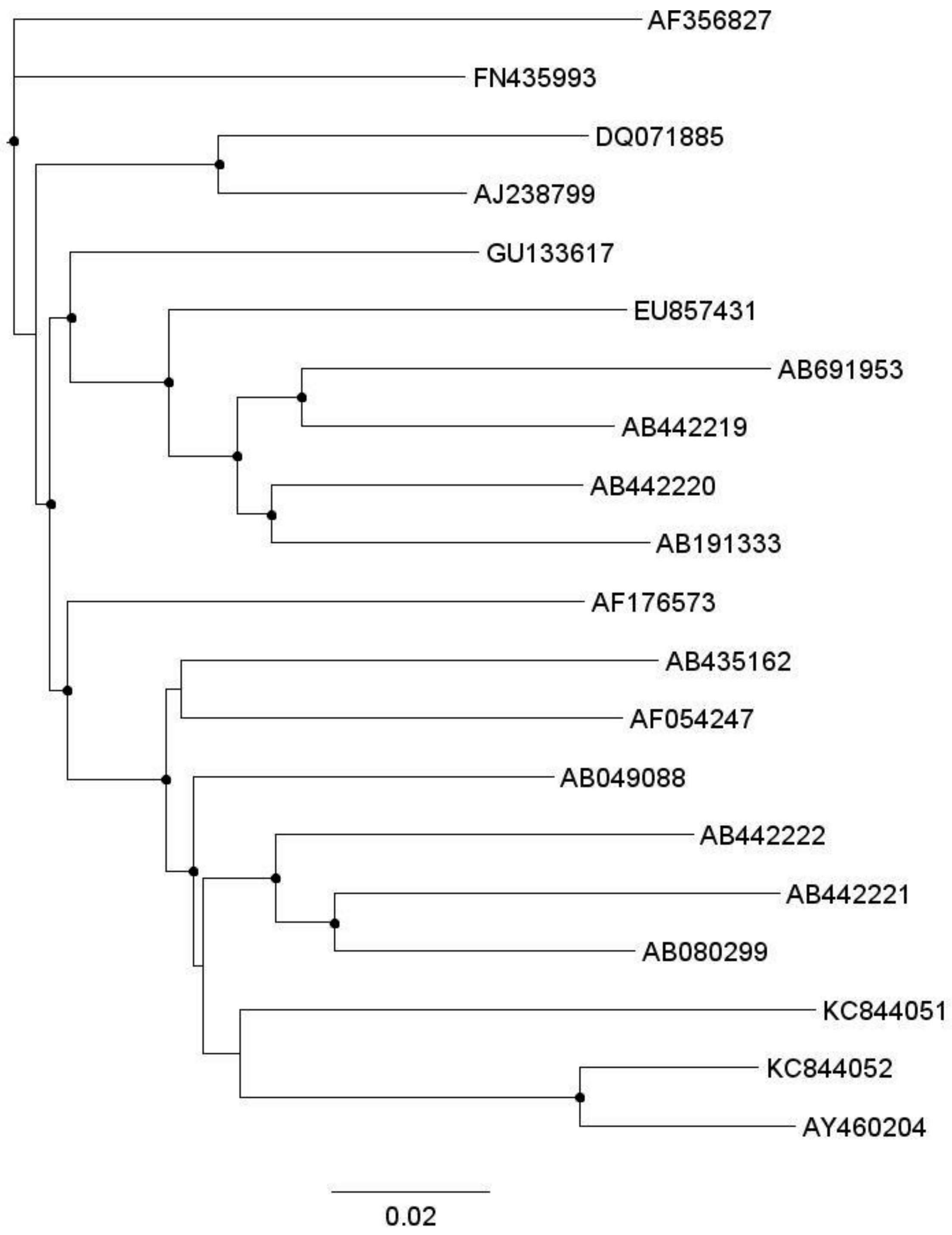

HCV-1b RNAalifold (mixed model)

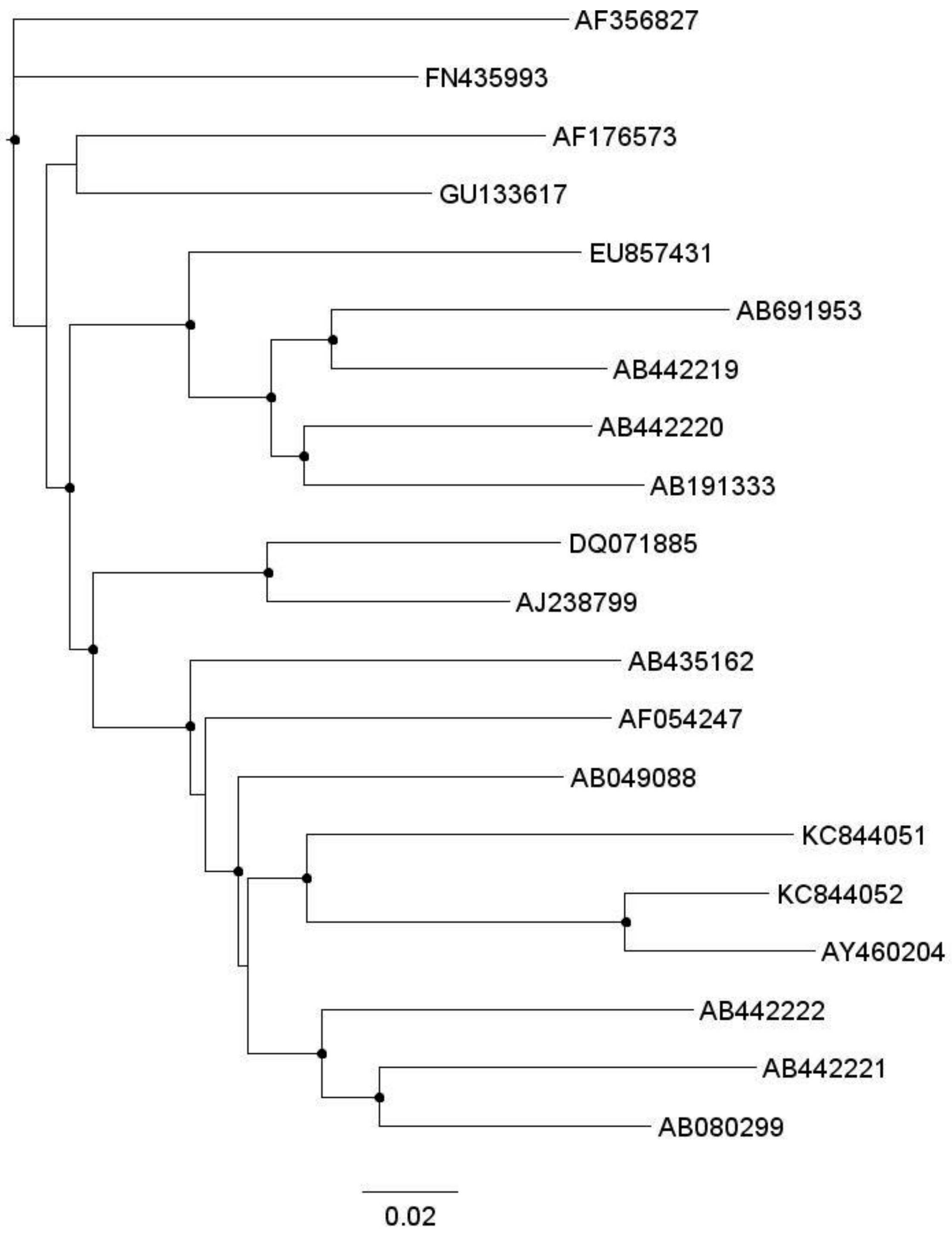

Supplementary Figure S.2 (cont)

HCV-1b SHAPE (DNA model)

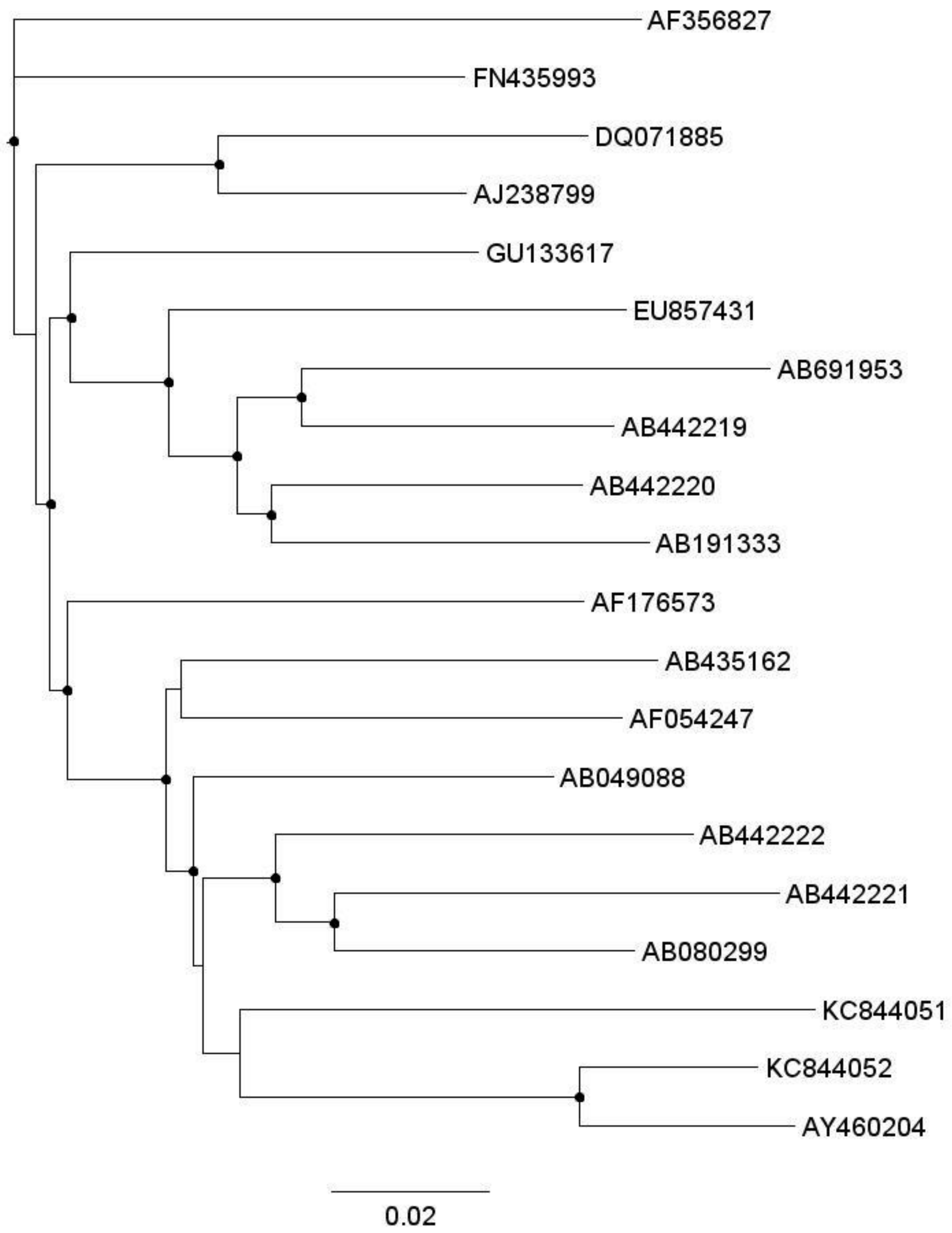

HCV-1b SHAPE (mixed model)

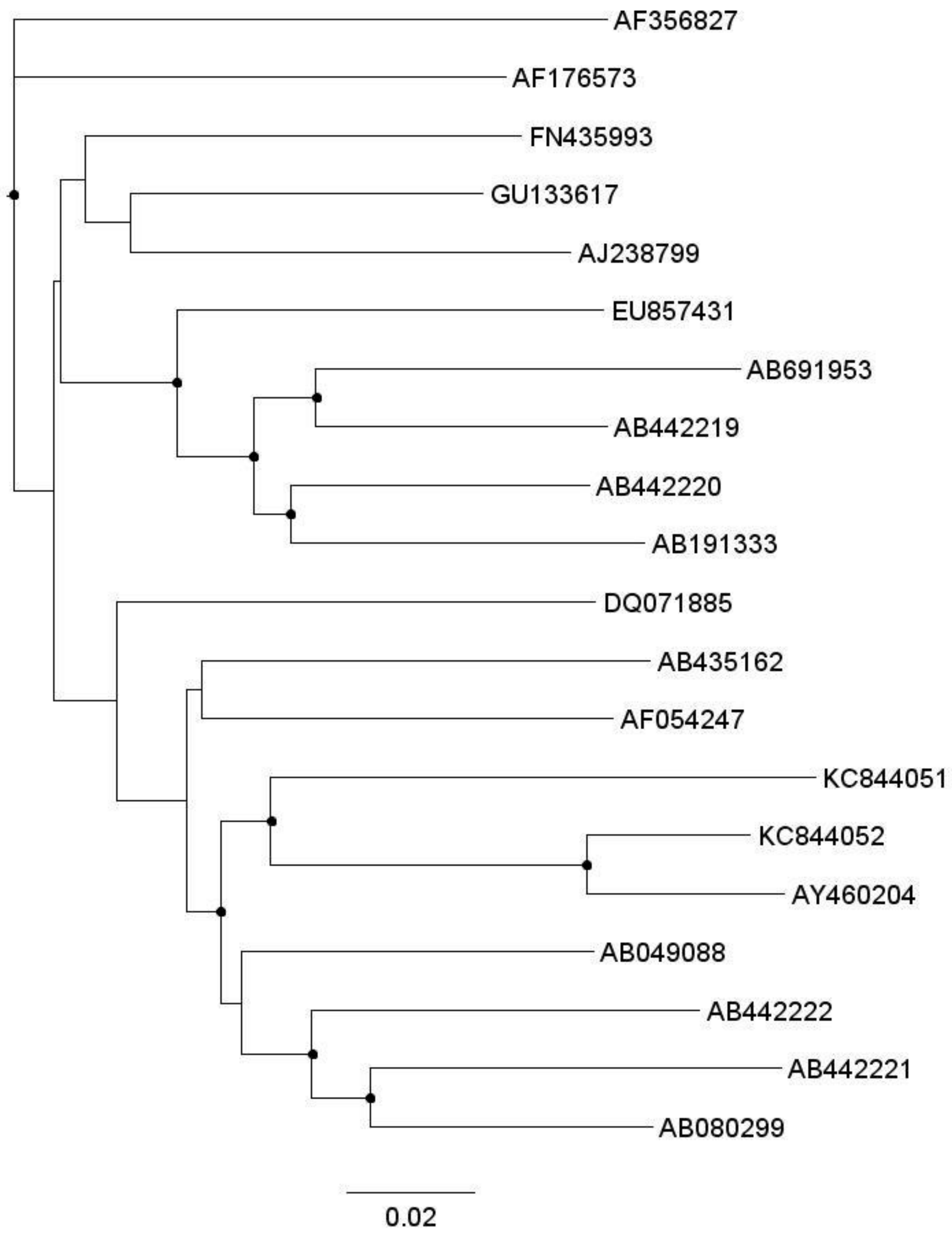

Supplementary Figure S.2 (cont)

HIV-1B RNAalifold (DNA model)

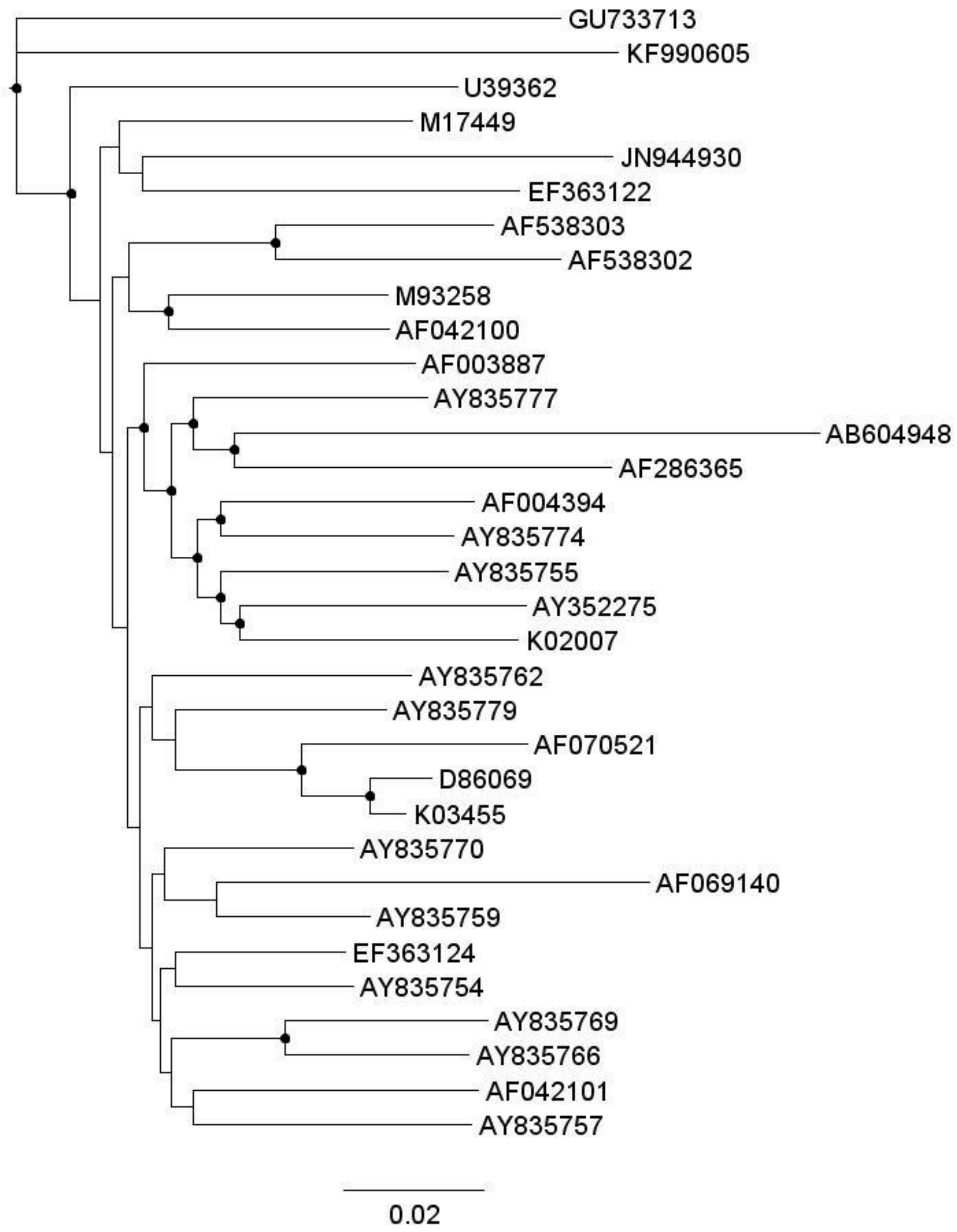

HIV-1B RNAalifold (mixed model)

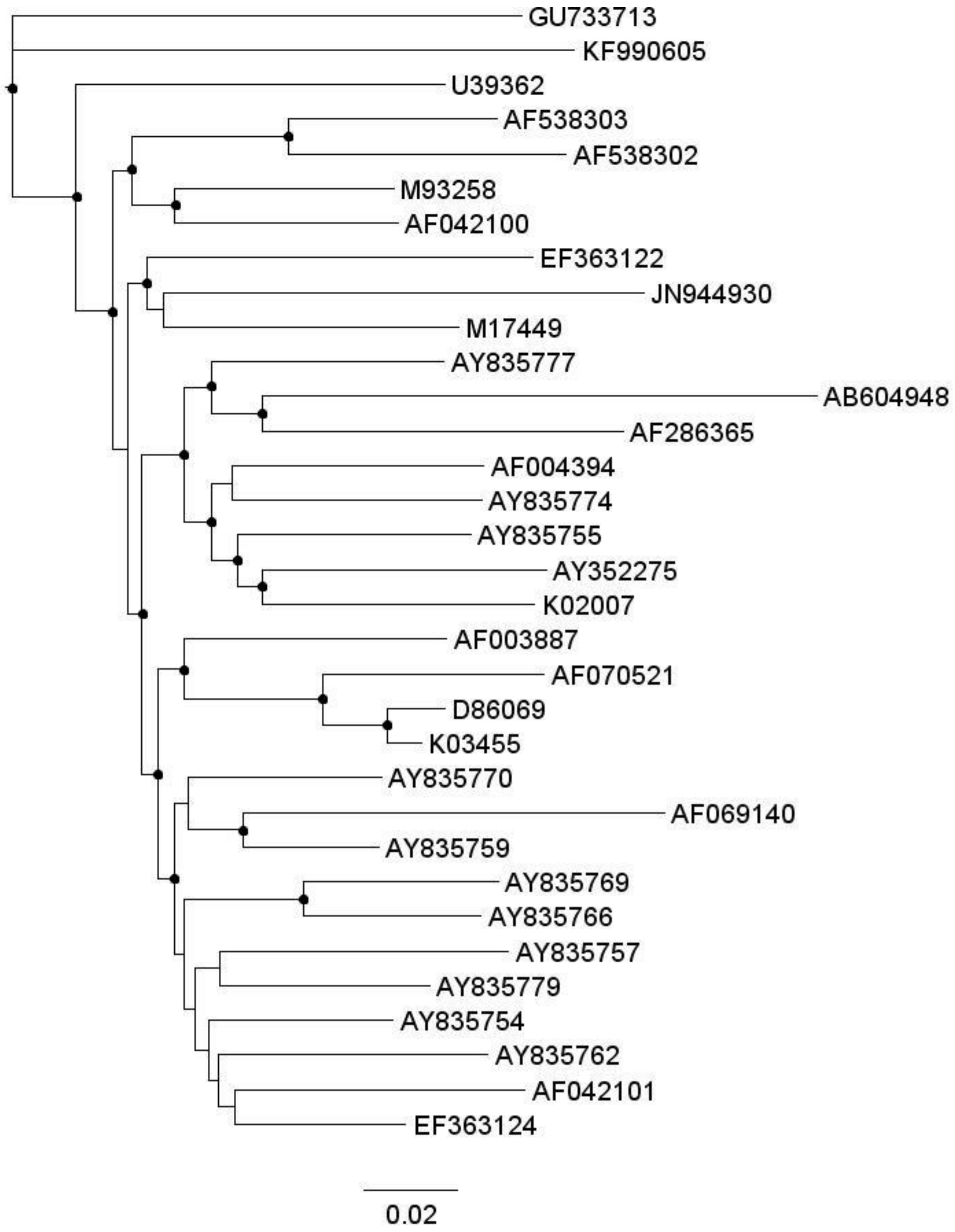

Supplementary Figure S.2 (cont)

HIV-1B SHAPE (DNA model)

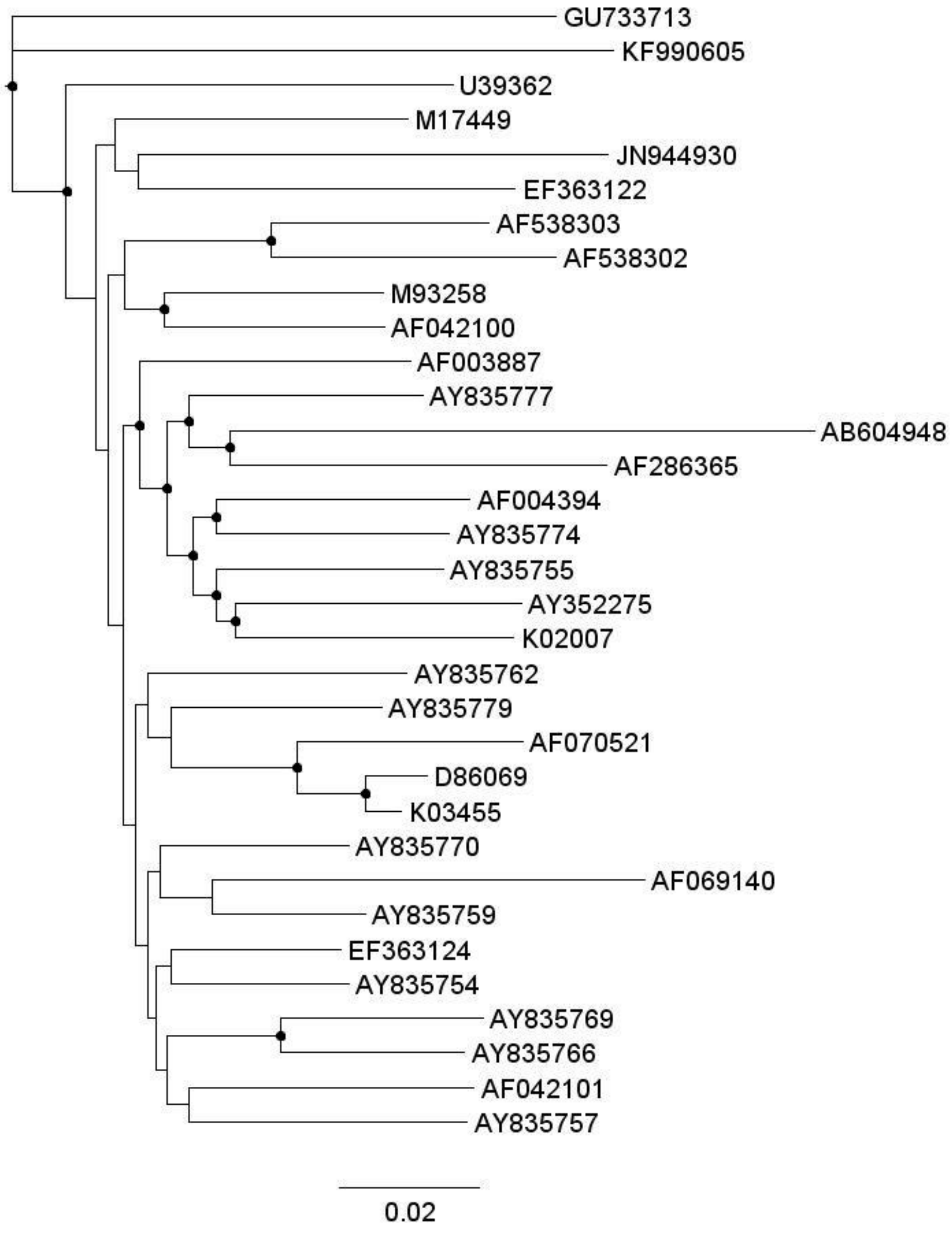

HIV-1B SHAPE (mixed model)

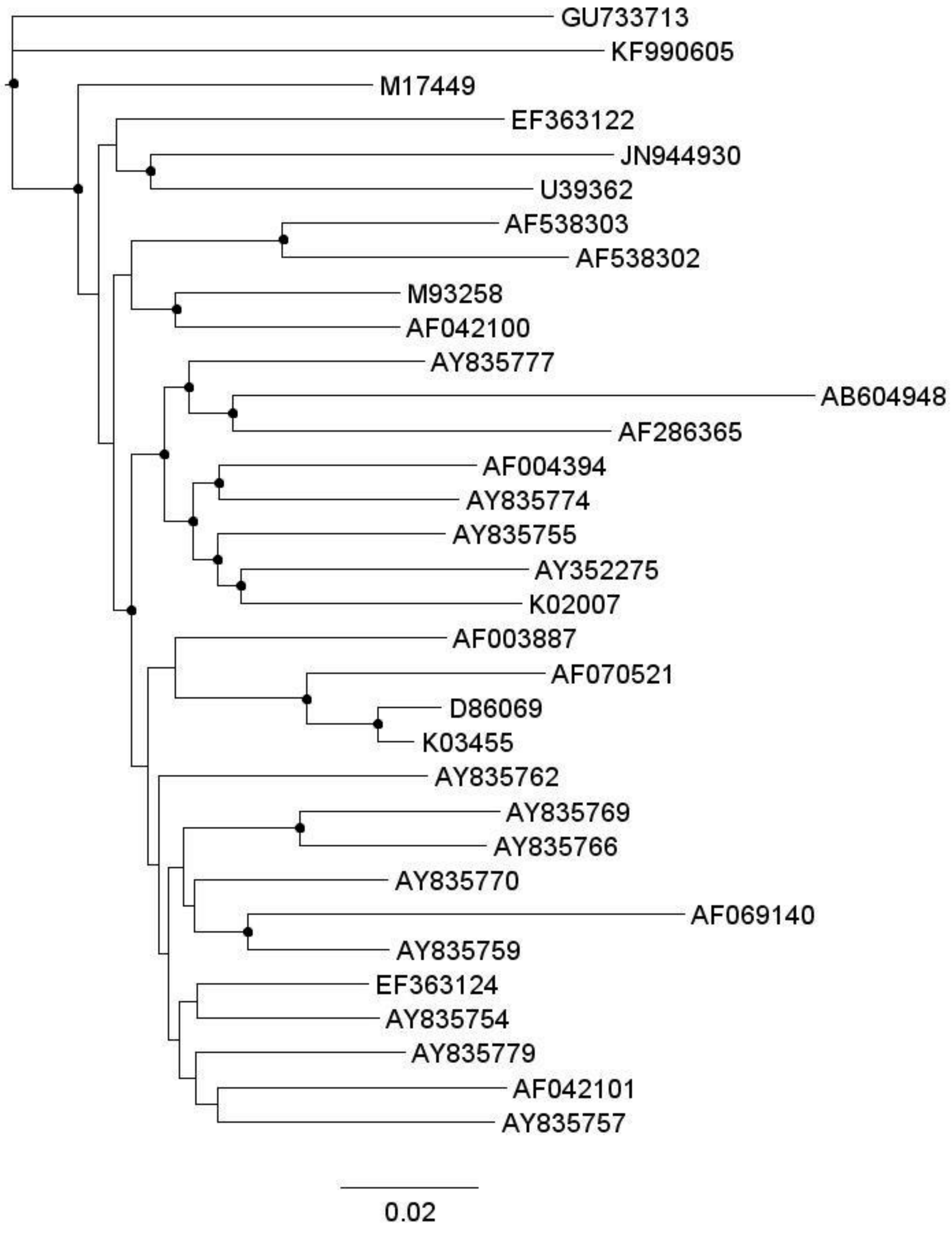

Supplement: Supplementary Figures [file evx273_supp.zip › SF2_trees.pdf]
